# An antisaccade task for measuring the attentional characteristics of social information processing in children with autism spectrum conditions

**DOI:** 10.1192/j.eurpsy.2021.606

**Published:** 2021-08-13

**Authors:** M. Lizarán, R. Sahuquillo-Leal, M. Perea, A. Moreno-Giménez, L. Salmerón, S. Simó, M. Vento, A. García-Blanco

**Affiliations:** 1 Neonatal Research Group, The Medical Research Institute Hospital La Fe (IIS La Fe), Valencia, Spain; 2 Department Of Psychology, University of Valencia, Valencia, Spain

**Keywords:** inhibitory control, autism spectrum condition, antisaccade task, eye-tracker

## Abstract

**Introduction:**

Autistic Spectrum Condition is often characterized by the presence of deficits in social interaction. An abnormal attentional processing may explain these difficulties, as it has been suggested that individuals with autism spectrum conditions may have problems with orienting attention to socially relevant stimuli and/or inhibiting their attentional responses to irrelevant ones.

**Objectives:**

The aim of the current study is to shed light on this issue by the assessment of the attentional orienting and inhibitory control to emotional stimuli (angry, happy, and neutral faces).

**Methods:**

An antisaccade task (with both prosaccade and antisaccade blocks) was applied to a final sample of 29 children with autism spectrum conditions and 27 children with typical development.

**Results:**

The main findings were: i) children with autism spectrum condition committed more antisaccade error when seeing angry faces than happy or neutral faces, while children with typical development committed more antisaccade errors when seeing happy faces than neutral faces, and ii) latencies in the prosaccade and antisaccade blocks were associated with the severity of autism symptoms.

**Conclusions:**

These results suggest that children with autism spectrum conditions show an impaired inhibitory control when angry faces are presented. This bias to negative high-arousal information is congruent with affective information-processing theories suggesting that threatening stimuli induce an overwhelming response in autism. From a clinical perspective, therapeutic strategies that focus on shifting attention to emotional stimuli may improve autism symptomatology and their socials functioning.

